# A personal perspective on medicinal and pharmaceutical chemistry

**DOI:** 10.3389/fchem.2014.00008

**Published:** 2014-02-27

**Authors:** Bimal K. Banik

**Affiliations:** Department of Chemistry, The University of Texas-Pan AmericanEdinburg, TX, USA

**Keywords:** medicinal chemistry, pharmaceutical chemistry, molecules, drugs, challenge

The search for new organic and inorganic compounds for treatment of different types of diseases is the aim of intense study. Over the past decades, many new compounds have been synthesized and isolated from numerous sources. However, only a fraction of them have demonstrated activity against human diseases. Despite the progress in research, the morbidity and mortality of several diseases including cancer, cardiovascular diseases, and HIV remains a major worldwide health problem. The effectiveness of currently available drugs is limited by high toxicities, low permeability, low half-life, and intrinsic or acquired drug resistance (Fesik, 2005; Gonzalez-Angulo et al., 2007). On this basis, it is crucial to search for novel molecules with high potency, low toxicity, and mutagenicity with selective activities that are able to overcome frequently developed resistance to available drugs. The challenging part in medicinal and pharmaceutical research is to differentiate between normal and affected cells and targets. For example, specific activation of cell death only in cancer cells without hampering normal healthy cells is one of the most difficult and hardest problems faced. However, this challenge opens up possibilities for conducting new medicinal and pharmaceutical research. Significant successes have also been realized in many research areas including: central nervous system, pulmonary system, oncology, infectious diseases, inflammation, cardiovascular, metabolism, and HIV. This has been possible because of the discovery of a number of new cellular targets including enzymes, synthetic pathways, novel reagents, development of new and target validation methods. Although inhibition of enzymes by small molecules and the identification of some of their targets have been confirmed, an improvement of the efficacy by structure-activity relationship studies using the computer-assisted data has failed in some instances. This is not surprising since many drugs can attack the diseased and normal cells in a variety of ways. For example, adriamycin was shown to be a DNA binding agent for many years. But, polymeric conjugates of adriamycin exert anticancer activity without entering into the cell nucleus (Tritton and Yee, 1982; Tritton, 1991). It was found that the drug-conjugate was located at the cell membrane and adriamycin was still bound to the polymer.

Our research group has been involved in the development of new anticancer agents which have not been explored systematically^1^. Our endeavor in this area has not been smooth and problem free because we have used polyaromatic amine derivatives as key components in our molecules. The main criticisms were that these molecules could generate mutagenicity in the cell. Extensive research has confirmed that certain polycyclic aromatic compounds are mutagenic and carcinogenic. Does this mean that all polycyclic aromatic compounds are mutagenic? The obvious answer is “no.” The fundamental organic compound benzene is carcinogenic, but several hundred benzene derived compounds are extremely useful drugs. Numerous anthracyclines, metal-containing molecules, and mustards are presently in clinical use.

The chemistry, biology, pharmacology, medicinal/pharmaceutical values of β-lactams are investigated extensively. A number of β-lactams that have been widely used in humans and animals are very safe antibiotics. We hypothesized that novel β-lactam drugs can be designed and synthesized which will have both an enhanced anticancer activity and low toxicity to normal tissue. We have designed, synthesized, and tested numerous β-lactams in racemic and chiral forms and have shown that many of them possess excellent antitumor activity *in vitro* against human cancer cell lines such as ovarian, skin, breast, colon, leukemia, prostate, and pancreatic cancer cell lines at low concentration *in vitro* and *in vivo* (Banik et al., 2003, 2004, 2005, 2010a,b,c; Bandyopadhyay et al., 2009, 2010, 2012; Bandyopadhyay and Banik, 2010; Banik and Becker, 2010a; Banik, 2011, 2012; Shaikh et al., 2011; Banik and Manhas, 2012; Shaikh and Banik, 2012). In some examples, the activity has exceeded that of cisplatin, a well-known anticancer drug. The active β-lactams block G_2_/M and G_0_/M checkpoints in cancer cell lines. Importantly, it has been observed that these β-lactams are less toxic to mice than some known clinically active drugs and were non-mutagenic when tested in bacterial and animal systems. This study may be used to develop translational science using β-lactam anticancer agents following cell-, animal- and gene-based approaches.

Despite an enormous body of work that describes the cellular reaction of polycyclic aromatic hydrocarbon (PAH), the application of these derivatives as anticancer agents has not been explored systematically. Our work in this area has culminated in the synthesis of a large number of planar molecules using highly lipophilic polycyclic aromatic amines as the nucleus for a systematic examination of modifications of structures that might result in selective interactions with cancer cells. This study has established lead compounds that are very effective against a variety of cancer cell lines. From structure-activity and *in vitro* and *in vivo* studies, lead compounds that are highly potent in HT-29 (human colon) and SKOV-3 (human ovarian) cell lines have been developed. They were shown to produce selective apoptosis in HL-60 cell lines. Although the major focus of therapeutic studies in cancer chemotherapy has been alterations of DNA replicative and repair functions, our studies have suggested that interactions with cell membranes may also play an important role in anticancer effectiveness^1^ (Becker and Banik, 1998; Becker et al., 2000; Banik and Becker, 2001a,b, 2010b; Banik et al., 2010d).

These explorations have taught me numerous lessons which I would like to share with. Unless we recruit dedicated and creative students, postdoctoral fellows and scientists in tackling some of the most challenging problems, there will be no progress made. Creativity does not really depend on the age although it has some influence on experience. In science classes the understanding of concepts and the ability to solve problems should be emphasized over memorization. Students should be encouraged to develop their own problem-solving strategies. In particular, organic, medicinal/biochemists/pharmacists have to know how to design a molecule, what reaction to perform, which reagents and experimental conditions to use during the development of medicinal drugs and pathways (how these drugs interact with the cell and cure medical disorders). Thus, problem solving is crucial in learning and practicing science. A researcher has to emphasize that research does not always proceed the way it is planned and that it is crucial to identify all the possibilities. This may turn out that the undesired products are more crucial than what we wanted to prepare. During the course of our investigation, we have not obtained results predicted by many significant researchers. It was difficult to convince the scientific community some of the unprecedented science. However, appropriate support from experiments, instrumental work, and evidences have greatly facilitate our research. We see lots of changes are going in the academic, industry and business areas. These changes may be necessary for the future prospect of our generations. One should keep in mind that this process should not dishearten bright scientists. No strong research program in chemistry and pharmacy can be built without the assistance and direct participation of creative scientists.

Out of 10,000 molecules synthesized or isolated for medicinal and pharmaceutical research, only one becomes clinically active per year. The entire process required 10–18 years. Why do we make such low progression despite our combined efforts with numerous sophisticated methods and tools? As scientists in medicinal and pharmaceutical research, it is our duty to explore our field with no set boundaries. Perhaps we are overlooking or overthinking crucial steps that are of importance and have medicinal value. Not all molecules that are designed or isolate have a planned biological activity. In many cases determining whether or not a molecule will exhibit any activity is a difficult process. We have little control over the activities of most of the molecules that are synthesized or isolated, but we do have control over the approach as to the discovery success. Every step is crucial for success, starting from the basics of drug design, synthesis, alteration of structure, and testing of molecules. It has been established by many groups that the most obvious route to discovery is often not the best. A compound designed for a particular disease may have other significant therapeutic applications. An identification of synergistic effects of two drugs and drug-drug interactions in living cells are very complex process. Although many patients take multiple drugs every day to remain healthy and this may prevent one-form of disease. How can we be certain that these multiple drugs would not demonstrate serious side effects which may be life threatening? Since many medicinal and pharmaceutical products have become our friends in the fight against diseases through non-traditional methods due to serendipity, we should follow the experiments carefully with dedication. Each step requires the best efforts capable of each scientist. Only as a whole are we capable of brightening our future in medicinal and pharmaceutical research.

## Footnotes

^1^http://portal.utpa.edu/utpa_main/daa_home/cosm_home/chem_home/chem_faculty/chemistry_banik
